# Emerging Frontiers in Neuro-Oncology: Insights into Extracellular Vesicle-Driven Tumor Mechanisms and Nanotherapeutic Strategies

**DOI:** 10.3390/ijms262411826

**Published:** 2025-12-07

**Authors:** Tommaso Colangelo, Anna Alessia Saponaro, Gianluigi Mazzoccoli, Gaetano Serviddio, Rosanna Villani

**Affiliations:** 1 C.R.E.A.T.E.—Center for Research and Innovation in Medicine, Department of Medical and Surgical Sciences, University of Foggia, 71122 Foggia, Italy; tommaso.colangelo@unifg.it (T.C.); anna.saponaro@unifg.it (A.A.S.); rosanna.villani@unifg.it (R.V.); 2 Unit of Cancer Cell Signalling, Fondazione IRCCS Casa Sollievo della Sofferenza, 71013 San Giovanni Rotondo, Italy; 3 Chronobiology Laboratory, Fondazione IRCCS Casa Sollievo della Sofferenza, 71013 San Giovanni Rotondo, Italy; g.mazzoccoli@operapadrepio.it; 4 Internal Medicine and Liver Unit, Department of Medical and Surgical Sciences, University of Foggia, 71122 Foggia, Italy

**Keywords:** extracellular vesicles (EVs), glioblastoma multiforme (GBM), microRNA, therapeutic vehicles, tumor aggressiveness

## Abstract

Brain tumors encompass a heterogeneous group of neoplasms, including primary and secondary metastatic lesions, with glioblastoma multiforme (GBM) representing the most aggressive primary malignancy. Despite advancements in surgical resection, radiotherapy, and chemotherapy, the prognosis for GBM remains poor due to its infiltrative nature, tumor heterogeneity and resistance mechanisms. Emerging diagnostic tools, such as liquid biopsies, and therapeutic strategies leveraging extracellular vesicles (EVs) are reshaping the field of neuro-oncology. EVs, lipid bilayer-enclosed particles secreted by cells, carry oncogenic cargo such as microRNAs and molecular chaperones, influencing tumor progression, immune evasion, and therapy resistance. Recent research highlights their potential as biomarkers for early diagnosis and vehicles for targeted drug delivery across the blood–brain barrier (BBB). EV-based nanotherapeutics show promise in improving treatment precision, reducing systemic toxicity, and advancing precision medicine in brain tumor management. However, challenges related to EV heterogeneity, cargo-loading efficiency, and large-scale production must be addressed to fully realize their therapeutic potential. This review explores the multifaceted roles of EVs in brain tumors, emphasizing their diagnostic, prognostic, and therapeutic applications.

## 1. Background

Brain tumors are a heterogeneous group of neoplasms originating within the central nervous system (CNS), classified into primary tumors and secondary metastatic lesions. Among primary CNS tumors, glioblastoma multiforme (GBM) is the most aggressive and prevalent primary malignancy in adults, characterized by a poor prognosis and a five-year survival rate of less than 10% [1,2]. These tumors represent a significant challenge in oncology, with an incidence rate of approximately 5–6 new cases per 100,000 individuals annually in Europe and the United States [3]. Globally, the most recent GLOBOCAN 2022 estimates indicate that brain and central nervous system tumors accounted for approximately 320,000 new cases and 245,000 deaths in 2022, with the highest burden observed in high-HDI regions such as Europe and North America [4].

Current therapeutic strategies include surgical resection, radiotherapy and chemotherapy, with temozolomide remaining the standard chemotherapeutic agent [2]. Surgical resection aims to maximize tumor removal while preserving neurological function, although the infiltrative nature of gliomas often precludes complete resection. Radiotherapy and chemotherapy target residual tumor cells, but their efficacy is frequently hindered by the blood–brain barrier and the heterogeneity of tumor cell populations [2]. Emerging treatments, including immunotherapies and targeted therapies, have shown promise across tumor types but face challenges such as resistance mechanisms and CNS-specific barriers like the blood–brain barrier [2,5]. This scenario highlights the need for innovative approaches that enable earlier and more precise diagnosis and improved patient monitoring and treatment compared with current approaches. Recently, liquid biopsies have emerged as a promising non-invasive alternative for identifying new molecular biomarkers released by the tumor. One key candidate are the extracellular vesicles (EVs), lipid bilayer-enclosed particles released by cells that facilitate intercellular communication [6]. EVs play a critical role in the pathogenesis of brain tumors, carrying oncogenic cargo such as microRNAs (miRNAs) and molecular chaperones. EVs facilitate tumor progression, immune evasion and therapeutic resistance, while also offering potential as biomarkers and drug delivery systems [7,8].

Nanotherapeutic strategies are being developed to exploit the unique properties of EVs, aiming to improve treatment precision and efficacy while minimizing systemic toxicity. This emerging field offers a pathway toward precision medicine in neuro-oncology, paving the way for innovative therapeutic advancements [1,9]. This review explores the multifaceted roles of EVs, highlighting their potential as diagnostic, prognostic and therapeutic biomarkers while underscoring the innovative potential of EV-based strategies in neuro-oncology.

## 2. Brain Tumors

### 2.1. Classification

Brain tumors include a range of neoplasms classified according to the World Health Organization (WHO) grading system for CNS tumors, which assigns a malignancy grade (from I to IV) based on a combination of histopathological criteria, immunohistochemical data, and molecular profiles [10,11,12]. Historically, primary CNS tumors were defined only by histological criteria. However, the 2016 revision of the WHO classification incorporated key molecular genetic alterations alongside classic histology, resulting in a combined phenotypic and genotypic diagnostic approach. By pairing histopathological names with genetic features (e.g., glioblastoma, IDH-wildtype), this system ensures more precise diagnoses, with molecular profiles considered more informative in case of discordance between histology and molecular findings [13,14]. This integrated approach improves diagnostic accuracy and objectivity, enabling better prognostic assessments and treatment planning [14,15].

According to the Central Brain Tumor Registry of the United States (CBTRUS), approximately 29.7% of all primary brain tumors are malignant and 70.3% are non-malignant, making non-malignant tumors more than twice as common as malignant ones [15].

Brain tumors can be further divided into primary, which originate directly within the brain, and secondary or metastatic lesions arising from extracranial malignancies [16,17,18,19]. Primary brain tumors represent approximately 2% of all cancers, with an overall annual incidence of 23 cases per 100,000 individuals, which increases with advancing age [15]. Among malignant entities, gliomas account for 70% of cases and are traditionally classified according to the presumed glial cell of origin into astrocytomas, oligodendrogliomas and ependymomas [13,16,17]. Glioblastoma (GBM) represents the most frequent and aggressive astrocytic tumor, characterized by marked inter- and intra-tumoral heterogeneity, with co-existing cancer stem cells, differentiated tumor cells, and non-neoplastic stromal components, including vascular cells, microglia and infiltrating immune cells [20,21,22]. This heterogeneity significantly complicates the investigation of GBM, which constitutes more than half of glioma cases, associated with higher malignancy grades and poor prognosis. In contrast, meningiomas are the most common primary non-malignant tumor, comprising 36% of all brain tumors, followed by pituitary tumors and nerve sheath tumors [13,16,17].

Regarding metastatic brain tumors, a wide range of extracranial malignancies capable of spreading to the CNS are described in the literature. Brain metastases are widespread in patients with lung and breast cancers. Non-small cell lung cancer accounts for approximately 60% of all metastatic brain tumors, while up to 30% of breast cancer patients also develop brain metastases [18,19,23]. Other common sources of brain metastases include melanoma, kidney, and colon cancers [18,24].

### 2.2. Risk Factors

Several risk factors for brain tumors have been established, although the majority of cases remain of unknown etiology. Less than 5% of primary brain tumors are associated with genetic predisposition syndromes, such as neurofibromatosis types I and II or other hereditary conditions; in comparison, familial cases account for approximately 5% of gliomas [25,26]. Ionizing radiation is the best-established environmental risk factor and increases the risk of both benign and malignant tumors, particularly when exposure occurs at a young age [16,26,27,28,29]. By contrast, non-ionizing radiation, such as radiofrequency electromagnetic fields (RF-EMF) from cell phone use, remains a topic of controversy, with current evidence not supporting a strong causal link [30]. The World Health Organization (WHO) and the International Agency for Research on Cancer (IARC) initially classified RF-EMF as “possibly carcinogenic to humans” (Group 2B) in 2011, and subsequently upgraded it to “probably carcinogenic” (Group 2A) in 2015 [31,32]. However, additional high-quality epidemiological and mechanistic studies are required to clarify their role in tumorigenesis. Interestingly, epidemiological data suggest an inverse association between allergic/autoimmune conditions and glioma risk, pointing to a role for systemic immune surveillance [33,34,35]. Overall, these data reinforce the concept that genetic susceptibility, DNA damage and immune dysregulation converge on brain tumorigenesis, mechanisms that are tightly connected to EV-mediated signaling and relevant to their roles in brain tumor biology.

### 2.3. Diagnosis and Limitations

The diagnosis of brain tumors, particularly GBM, is one of the most complex challenges in neuro-oncology due to the tumor’s aggressive progression and extensive inter- and intra-tumoral heterogeneity [36]. Currently, clinical practice relies on imaging modalities such as Gadolinium-enhanced magnetic resonance imaging (MRI), considered the gold standard for detecting intracranial tumors [37,38,39]. MRI allows detailed characterization of tumor boundaries and vascularity through multimodal approaches like diffusion tensor imaging, MR perfusion, and MR spectroscopy [38,40,41]. However, despite its utility, MRI has significant limitations. Its spatial resolution cannot detect microscopic tumor invasiveness and differentiate a true tumor progression from a pseudo-progression, a misdiagnosis that occurs in up to 36% of cases [42,43,44]. In patients unable to undergo MRI, alternatives like computed tomography (CT) are used, although they offer reduced resolution and are less effective in assessing posterior fossa lesions [37]. These imaging gaps can delay appropriate treatment adaptations and obscure real-time disease dynamics.

Beyond imaging, histopathological examination of biopsied tissue remains fundamental for diagnosis and molecular profiling. However, biopsy-based approaches face substantial challenges, including their invasiveness, the inability to capture the full molecular heterogeneity of a tumor, and their temporal limitations, as they only provide a static snapshot of a dynamic disease [36,45]. Moreover, repeated biopsies to monitor disease evolution are impractical due to ethical concerns and procedural risks. This diagnostic bottleneck is further worsened by the lack of specificity in current methods and, in particular, by the highly infiltrative nature of GBM, which often leaves undetected residual tumor cells beyond the sampled region [44]. Moreover, even if significant efforts have been made to develop new therapeutic strategies, such as surgical procedures, radiotherapy, chemotherapy, and immunotherapy, brain tumors remain a major cause of morbidity and mortality worldwide [15,46]. This challenge is further exacerbated by late-stage diagnoses and the emergence of resistance to anticancer treatments [47].

Although imaging and tissue biopsy remain essential components for brain tumor diagnosis, liquid biopsy technologies, particularly those based on EVs, are emerging as ideal candidates for advancing diagnostic approaches [45,48]. Identifying specific biomarkers that could be detected using noninvasive methods may allow early diagnosis and real-time disease monitoring, including controlling the response to treatment and supporting the development of personalized therapeutic strategies.

## 3. Extracellular Vesicles: Biogenesis, Composition and Physiological Roles

EVs are a heterogeneous group of lipid-bound membrane particles that cannot replicate independently. They are synthesized and secreted by different cell types into the extracellular environment [49]. Initially, EVs were thought to function merely as a cellular mechanism for disposing of unwanted materials, overlooking their critical role as specialized mediators of intracellular communication [50]. These vesicles contribute not only to normal physiological processes but also to the progression of pathological conditions, making them an increasingly important focus of research [51].

According to the guidelines of the International Society for Extracellular Vesicles (ISEV), the term “extracellular vesicles” is preferred as a general descriptor for all secreted vesicles, due to the lack of consensus on identifying specific markers to differentiate between EV subtypes. Furthermore, EVs should be classified using operational terms based on size, density, molecular composition, or cellular origin, rather than biogenesis pathways, which are often challenging to determine [49].

EVs are broadly classified into three main subclasses: exosomes (EXOs), microvesicles (MVs), and apoptotic bodies [52]. EXOs, with 50–150 nm diameters, originate from the cell’s internal compartments to form early endosomes, which subsequently fuse with the plasma membrane to release their contents into the extracellular space [53,54]. MVs, ranging from 100 to 1000 nm in diameter, are generated through the outward blebbing of the plasma membrane via a regulated process that remains only partially understood. This process is influenced by cytoskeletal components and is highly dependent on the cell’s physiological state and surrounding microenvironment [53,54,55]. Apoptotic bodies, on the other hand, range from 100 nm to 5 μm and are released by cells undergoing programmed cell death [53]. Recently, additional EV subtypes, including migrasomes, large oncosomes and exophers, have been described, further emphasizing the heterogeneity of these vesicles [49,56,57,58]. In line with the most recent Minimal Information for Studies of Extracellular Vesicles (MISEV) 2023 recommendations, we preferentially use the operational term “EVs” for heterogeneous or mixed vesicle preparations and specify “small EVs (sEVs)”, “EXOs” or other subtypes only when the original studies provide clear biogenesis or marker-based evidence for such classification.

EVs can be isolated using several methods from a wide range of biological fluids, including blood, urine, saliva, breast milk, synovial fluid, tears, amniotic fluid, lymph, bile, and cerebrospinal fluid (CSF), making them highly accessible for clinical application [53,59,60,61,62]. Differential ultracentrifugation is considered the gold standard method for EV isolation. This technique separates EVs based on size and density by employing varying centrifugation speeds, enabling high-purity isolation. Its performance can be further improved by combining it with other approaches, such as density gradients or filtration, to enhance specificity and efficiency. Other methods, such as size-exclusion chromatography and immuno-isolation, are also commonly used, even if additional techniques and their combinations are actively being researched and validated [49,63,64].

EVs carry diverse cellular cargo derived from their parental cells, including proteins (e.g., cell surface receptors, signaling proteins, transcription factors, and enzymes), lipids, and nucleic acids (e.g., miRNA, mRNA, and DNA) [6,65]. Their surface is enriched with tetraspanin proteins (CD9, CD63, CD81, and CD82), which are involved in cell membrane interaction, invasion, and fusion. EVs also contain proteins essential for maintaining cellular homeostasis and protecting cells from stress or apoptosis, such as heat shock proteins (Hsps), including Hsp60, Hsp70, and Hsp90, which are also commonly used as vesicular markers [49,66,67]. By transferring this molecular cargo, EVs play a pivotal role in intercellular communication, facilitating molecular exchange. Additionally, they act as bioactive carriers of components that reflect the characteristics of their cell of origin, providing valuable insights into cellular interactions in both physiological and pathological contexts [68,69].

## 4. The Dual Roles of Extracellular Vesicles in Brain Tumor Biology: Friends or Foes?

Despite their various physiological roles, recent studies increasingly associate EVs with pathological conditions, including cancer, underscoring their significance in elucidating disease mechanisms. EVs are secreted by all cell types in the tumor microenvironment (TME), including cancer cells, and play pivotal roles in tumor progression. Cancer cells produce EVs in higher quantities and with more specific cargo than normal cells, reflecting their essential role in survival and malignancy [70,71]. Furthermore, EVs carry nucleic acids and proteins protected from degradation, making them more stable than circulating tumor cells (CTCs), cell-free DNA (cfDNA), and proteins [72]. These vesicles collaborate with secreted factors and direct cell-to-cell interactions, reprogramming normal cells in the TME to adopt tumor-supportive phenotypes. This dynamic interplay underscores EVs’ role as key mediators of cancer growth, invasion, and therapy resistance [1,6,73].

**Figure 1:**
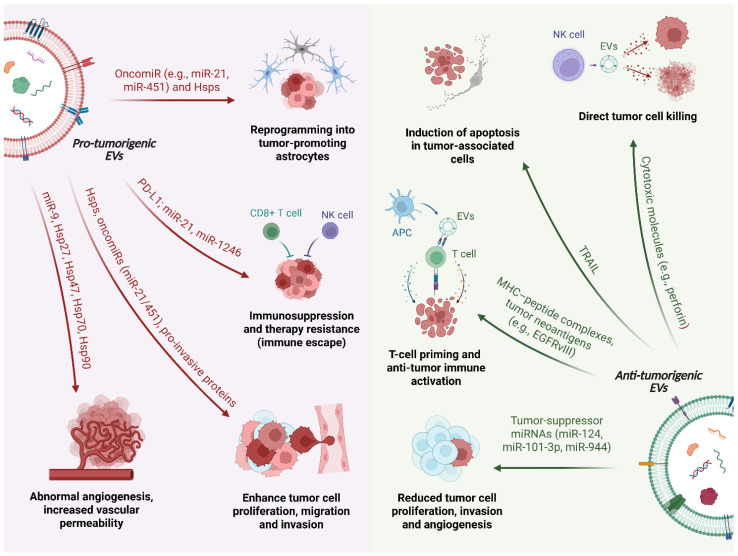
Dual roles of extracellular vesicles (EVs) in brain tumor biology. Schematic representation of the pro-tumorigenic (left) and anti-tumor/protective (right) functions of extracellular vesicles (EVs) in the brain tumor microenvironment. In the figure, a single EV is intentionally depicted as a split schematic, with its left and right halves representing pro-tumorigenic and anti-tumor/protective EVs, respectively. On the pro-tumorigenic side, glioblastoma cells release EVs enriched in heat shock proteins (Hsps), oncomiRs (e.g., miR-21, miR-451, miR-9) and immunomodulatory molecules such as programmed death-ligand 1 (PD-L1) and transforming growth factor-β (TGF-β). These vesicles promote abnormal angiogenesis and increased vascular permeability by reprogramming brain endothelial cells, enhance tumor cell proliferation, migration and invasion through Hsps and pro-invasive proteins, and reprogram astrocytes into tumor-promoting cells. In parallel, EVs act on immune cells to drive immunosuppression and therapy resistance by polarizing microglia/macrophages and inducing dysfunction of cytotoxic lymphocytes (CD8^+^ T cells, NK cells). On the anti-tumorigenic side, EVs derived from microglia and other brain cells can deliver tumor-suppressor miRNAs (e.g., miR-124, miR-101-3p, miR-944) to glioma cells, thereby reducing their proliferation, invasion and angiogenic potential. EVs released by NK cells carry cytotoxic molecules that mediate direct tumor cell killing, whereas EVs derived from antigen-presenting cells (APCs) transport MHC-peptide complexes and tumor neoantigens (such as EGFRvIII), contributing to T-cell priming and anti-tumor immune activation.

Focusing on the role of EVs in primary brain tumors, brain tumor–derived EVs (mainly small EXOs) regulate gene expression through surface signaling and by transferring their cargo to nearby and distant cells. Tumor cells exploit the information carried by EVs by releasing them into the CSF and bloodstream, influencing gene activity, facilitating communication across the body, and reprogramming normal cells within the TME to support tumor growth and invasion [74,75]. Upon being released into the extracellular space, EVs can engage in local autocrine or paracrine signaling, influencing nearby tumor cells or nervous system cells (e.g., astrocytes). Alternatively, they can cross the blood–brain barrier (BBB) and reach distant cells via systemic circulation. In either scenario, EVs deliver their cargo into the cytoplasm of recipient cells by fusing with their membrane. The cargo of EVs reflects the characteristics of the cells they originate from [76]. As a result, molecules such as miRNAs (e.g., miR-1) and Hsps, typically produced by tumor cells, may be selectively packaged into EVs and influence the tumor microenvironment and distant tissues (Figure 1 and Table 1) [76,77,78].

Consistent with MISEV2023, we use the term “EVs” as an umbrella term when the original studies do not clearly distinguish between vesicle subtypes, and we explicitly refer to “sEVs” or “EXOs” only when such distinction is made.

### 4.1. Pro-Tumorigenic Roles of EVs in Brain Tumors

In brain tumors, most tumor-derived EVs act as pro-tumorigenic mediators (Figure 1). Through the transfer of oncogenic proteins, Hsps and miRNAs, these vesicles sustain angiogenesis, support tumor growth and invasion, modulate the immune microenvironment toward an immunosuppressive state and favor resistance to therapy (Table 1).

Hsps predominantly act as pro-tumorigenic factors in the context of brain tumors (Table 1). For instance, Hsp27 [79,80], Hsp 47 [81,82], Hsp70 [83,84], and Hsp90 [85,86] have been detected on the surface of brain tumor-derived EVs, highlighting their potential utility as tumor biomarkers [99,100]. However, despite molecular chaperones being recognized as key contributors to brain tumor biology, limited data are available in the current literature on the specific roles of extracellular Hsps in these tumors. In contrast, miRNAs are more abundant in EVs than other cargo molecules [101]. Many of these EV-associated miRNAs function as oncomiRNAs, promoting tumor cell proliferation, migration, angiogenesis and immunosuppression (Table 1). Their effects are mediated by the post-transcriptional regulation of cell cycle-related factors and are proportional to the levels of the relevant miRNA [102,103].

In the context of GBM, the most common primary malignant brain tumor, in vivo studies using human plasma samples have revealed that a single GBM cell can secrete approximately 10,000 EVs within 48 h. These vesicles, enriched with the CD9 surface antigen, perform various functions in GBM progression and its interaction with the microenvironment [104,105]. For example, GBM-derived EVs, including EXOs, promote vascularization by reprogramming endothelial cells, leading to abnormal angiogenesis. Specific miRNAs, such as miR-9, are carried within these vesicles and downregulate angiostatic genes, facilitating the formation of new blood vessels that support tumor growth [87,88,89]. Additionally, EVs mediate communication between GBM cells and astrocytes, the most abundant glial cells in the brain. Through EVs release, GBM cells transform astrocytes from a protective role into reactive, tumor-promoting astrocytes, enhancing their growth and invasiveness. This process involves MYC pathway activation and p53 pathway inhibition, leading to increased secretion of pro-inflammatory molecules and matrix-degrading enzymes, which support tumor progression [106,107,108]. EVs also enhance the immunosuppressive activity of microglia. When microglia take up miR-451 and miR-21, highly abundant in GBM-derived EVs, their gene expression is altered, reducing their ability to respond to tumor cells and ultimately aiding tumor growth and progression (Table 1) [90,91,92].

Beyond promoting GBM cell survival and invasiveness via specific EV proteins (e.g., annexin A1, actin-related protein 3, integrin-β1, insulin-like growth factor 2 receptor, and Alix) [109], GBM-derived EVs play a significant role in creating an immunosuppressive environment that supports tumor growth and resistance to therapy. These GBM-derived EVs contain immunosuppressive molecules such as PD-L1 and TGF-β [110,111], as well as miRNAs (e.g., miR-10a [93], miR-21 [90,91,92], miR-29a [94], and miR-1246 [95]) that inhibit immune cell activity [92]. GBM-derived EVs modulate immune cells like microglia, monocytes, and dendritic cells, inducing tumor-supportive phenotypes, disrupting T-cell responses, and promoting tumor progression [112]. Moreover, they modulate cytokine secretion (e.g., VEGF and IL-6) and impair antigen presentation, thereby facilitating immune evasion and contributing to GBM persistence and resistance to therapy [113,114].

### 4.2. Anti-Tumorigenic and Protective Roles of EVs in Brain Tumors

Although most tumor-derived EVs support brain tumor progression, some brain-cell-derived EVs demonstrate a tumor-suppressive potential (Figure 1 and Table 1) [115]. For instance, microglia-derived sEVs containing miR-124 can reprogram GBM cells to reduce harmful metabolites, restoring central nervous system balance [96], while exosomal miR-101-3p [97] and glioma stem cell-derived exosomal miR-944 [98] can inhibit tumorigenesis by suppressing pathways essential for tumor growth. In oligodendroglioma, EVs containing Tumor Necrosis Factor-Related Apoptosis-Inducing Ligand (TRAIL) trigger apoptosis in astrocytes and neurons, modulating tumor–host interactions [116,117]. Additionally, EVs released by natural killer (NK) and endothelial cells display anti-tumor properties through the delivery of cytotoxic molecules and the suppression of inflammatory signals, respectively [118,119,120].

Emerging evidence further suggests that EVs secreted by antigen-presenting cells (APCs), such as B lymphocytes and dendritic cells, possess intrinsic immunogenic properties. These vesicles carry MHC molecules capable of inducing T-cell responses. Notably, EVs harboring tumor-specific neoantigens, such as EGFRvIII in GBM, can directly prime T lymphocytes or enhance dendritic cell-mediated cross-presentation to naive T cells [121,122,123]. GBM-derived EVs may also deliver tumor-associated antigens, providing potential targets for mRNA-based vaccination strategies.

Overall, these observations illustrate that EV-associated miRNAs and other EV cargos exert highly context-dependent functions in brain tumors. Apparent contradictions between pro- and anti-tumor roles can often be explained by differences in the cellular origin of EVs (e.g., tumor cells versus immune or stromal cells), the genetic and molecular background of the tumor (including distinct oncogenic drivers or mutational profiles), and the experimental models used (in vitro co-culture systems, orthotopic versus heterotopic in vivo models, or patient-derived samples). In addition, miRNAs are rarely delivered in isolation: EVs transport complex combinations of miRNAs, proteins and other non-coding RNAs, so that the net biological outcome reflects the balance between multiple, sometimes opposing, signals and the specific repertoire of targets expressed in recipient cells. Thus, the same miRNA may support tumor growth when enriched in tumor-derived EVs acting on endothelial or immune cells, yet exert tumor-suppressive effects when delivered in a different EV context or to a different target cell population. Recognizing this context dependency is essential for interpreting EV-miRNA data in neuro-oncology and for rationally exploiting EV-associated miRNAs as biomarkers or therapeutic agents.

From a translational perspective, additional layers of complexity arise when moving from reductionist models to patient-derived biofluids and in vivo settings. In plasma, serum or CSF, EVs released by malignant cells are extensively intermixed with vesicles originating from platelets, leukocytes, endothelial and neural cells, making it challenging to unequivocally assign specific molecular signatures or functional effects to the tumor-derived compartment without rigorous enrichment strategies or single-EV analytical approaches [43,45,99,104]. Moreover, EVs used as therapeutic vehicles are unlikely to remain static once administered in vivo: exposure to circulating proteins and to the TME can remodel their surface composition (including the formation of a “protein corona”), uptake pathways and immunological properties, potentially altering their behavior compared with that observed in vitro [104,124,125]. These considerations further underscore the need to validate EV-mediated tumor-promoting and tumor-suppressive activities in physiologically relevant models and to carefully account for EV heterogeneity when designing EV-based biomarkers and therapies.

Collectively, these context-dependent effects need to be carefully considered when interpreting EV-based biomarkers in clinical samples and when designing EV-centered therapeutic strategies for brain tumors.

## 5. EVs as Therapeutic Vehicles

The CNS presents several challenges for effective drug delivery to the brain, as drugs must overcome multiple barriers to reach therapeutic levels. Under physiological conditions, two main barriers protect the brain: the blood–brain barrier (BBB) and the blood-cerebrospinal fluid barrier (BCSFB). In the context of brain tumors, a third, pathological barrier, the blood-brain tumor barrier (BBTB), emerges, further complicating drug delivery.

The BBB regulates brain function by separating the CNS from peripheral circulation and protecting the brain from harmful substances. It comprises tightly packed endothelial cells along brain capillaries and selectively allows water, nutrients, and hydrophobic molecules to pass. The BBB protects the brain by actively removing lipid-soluble toxins and bacteria through P-glycoprotein-mediated efflux [126,127]. Most drugs cannot cross the BBB, limiting their therapeutic effectiveness for neurological conditions. Moreover, the normal functions of the BBB are disrupted in various brain diseases, including brain tumors. Gliomas, particularly high-grade ones, weaken the BBB by enhancing blood vessel growth and producing vascular endothelial growth factor (VEGF), leading to increased permeability [128]. However, this formation of blood vessels also leads to a malfunction of the BBB, creating a BBTB with altered functions. This disruption is partly due to poorly differentiated astrocytes that fail to support proper BBB function. The loss of claudins and occludins, proteins that maintain tight junctions in endothelial cells, also contributes to the leakage of the BBB [129,130,131]. Furthermore, brain tumors secrete substances like VEGF and cytokines that further damage the BBB, hindering the effectiveness of chemotherapy by preventing adequate drug delivery to tumor [132]. Therefore, new therapeutic approaches are currently under development and investigation, emphasizing employing endogenous cells and mechanisms for brain tumor therapy.

**Figure 2:**
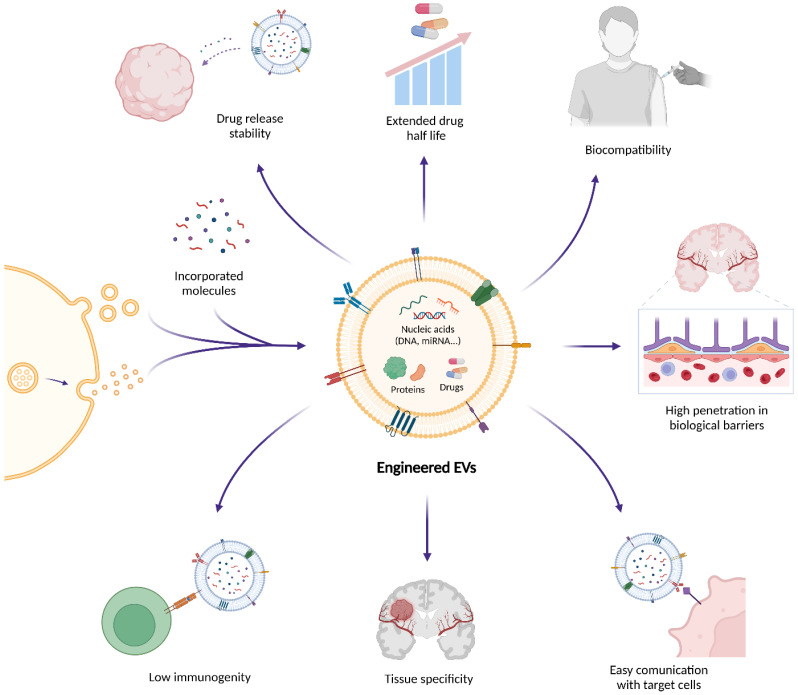
Therapeutic potential of engineered extracellular vesicles (EVs) in brain tumors. Extracellular vesicles (EVs) are emerging as therapeutic delivery systems that can encapsulate and transport bioactive molecules such as nucleic acids, proteins, and small drugs across biological barriers like the blood–brain barrier (BBB). Their natural biocompatibility and targeting specificity reduce the risk of adverse immune reactions and off-target effects. In addition, EV surfaces can be engineered through ligand or peptide display, genetic fusion of targeting motifs (e.g., Lamp2b-based constructs), or chemical conjugation strategies to enhance receptor-mediated uptake and improve and improve the interaction with the BBB and the blood–brain tumor barrier (BBTB). These modifications increase therapeutic efficacy and promote selective communication with target cells. Owing to these properties, EVs enable efficient drug delivery, immune modulation, and tumor targeting, positioning them as innovative platforms for next-generation precision therapies, immunotherapies, and RNA-based treatments in oncology and neuro-oncology.

Due to their intrinsic biological advantages, EVs have garnered significant attention as therapeutic delivery systems since they are excellent vehicles with biocompatibility, low immunogenicity, and the ability to carry functional cargo such as proteins, RNA, and small molecules across biological barriers like the BBB (Figure 2) [133]. EVs are also naturally present in biological fluids, making them a versatile and ubiquitous tool for therapeutic applications. Their targeting capabilities rely on receptor-ligand interactions, enabling precise delivery to specific cell populations, such as cancer cells [114]. These characteristics have been harnessed in various studies, mostly by using a common in vitro technique which involves passive mixing of the drug (e.g., curcumin) with isolated EVs [134,135], or active drug loading using sonication, incubation or electroporation [135,136,137].

Importantly, several EV-based approaches have provided in vivo proof-of-concept that these vesicles can at least partially overcome the BBB and BBTB. In preclinical glioblastoma models, brain endothelial cell-derived EVs loaded with VEGF siRNA were able to reach intracranial tumors after systemic administration and inhibit tumor-induced angiogenesis [138]. Similarly, Angiopep-2/TAT dual-modified EVs carrying CRISPR-Cas9 components efficiently crossed the BBB, accumulated in glioblastoma lesions and achieved gene editing of glutathione synthetase, thereby sensitizing tumors to radiotherapy [139]. In another study, mesenchymal stromal cell-derived EVs loaded with the tumor-suppressive miR-1208 were shown to cross the BBB and suppress glioma progression, an effect further enhanced by focused ultrasound [140]. Together, these findings support the notion that native and engineered EVs can exploit endogenous transport pathways or be further functionalized to penetrate the BBB/BBTB and deliver therapeutic cargo to brain tumors, although issues such as delivery efficiency, off-target distribution and safety still represent important challenges for clinical translation.

### 5.1. Loading EVs with Functional Cargo

From a drug-delivery perspective, EVs can be viewed as natural nanocarriers whose lumen and membrane can be loaded with bioactive cargo. Therapeutic molecules can be incorporated into EVs using two main approaches: pre-loading and post-loading methods (Table 2). Recent studies have further expanded these loading approaches by introducing advanced EV engineering strategies, including membrane functionalization, hybrid EV-nanoparticle designs, and genetically encoded cargo-sorting systems, with more precise control over targeting, pharmacokinetics and cargo release [141,142].

In pre-loading approaches, drugs are introduced into the parental cells, which subsequently release drug-enriched EVs (Table 2) [136]. This strategy preserves EV membrane integrity and can support continuous production. The two most common pre-loading techniques are co-incubation and genetic transfection. Co-incubation is conceptually simple and particularly suitable for lipophilic drugs such as doxorubicin or paclitaxel, but typically achieves relatively low loading efficiency [136,137,143]. By contrast, transfection allows the overexpression of defined nucleic acids (e.g., siRNA, mRNA) or proteins that are then packaged into or displayed on EVs, providing more precise control over cargo composition. However, its success depends on transfection efficiency and cell viability and offers limited control over the exact number of cargo molecules per vesicle [124,136,137,144].

Post-loading, by contrast, involves drug incorporation after EV isolation and offers greater customization (Table 2). Passive loading relies on simple diffusion through co-incubation, but often suffers from low efficiency that might depend on the lipophilic properties of the drug and the concentration gradient [134,136,137]. On the other hand, active loading by using physical or chemical methods improves cargo entry by temporarily enhancing EV membrane permeability. Physical induction, such as electroporation or ultrasonication, generally involves the rapid disruption of EV membranes using external forces, with the success of the drug-loading depending on the recovery of the membrane integrity of the EVs [136,137,145]. Chemical induction employs transfection agents to enhance cargo loading without compromising the integrity of the EV membranes [136,145]. Emerging chemical approaches, such as saponin treatment and calcium chloride-mediated transfection, have improved efficiency compared to traditional techniques like electroporation, being simpler and more stable [146,147]. Each method presents trade-offs between efficiency, structural integrity, and practical feasibility, offering versatile options for developing therapeutic EV-based delivery systems.

**Table 2:** Comparison of pre-loading and post-loading strategies for loading therapeutic cargo into extracellular vesicles (EVs).

Loading Strategy	Techniques	Typical Cargo	Advantages	Limitations	References
Pre-loading	Co-incubation of parental cells with free drug	Lipophilic small-molecule drugs (e.g., doxorubicin, paclitaxel)	Simple; no EV isolation; preserves EV membrane; suitable for continuous production	Low and variable loading; mainly for lipophilic drugs; depends on cell uptake and viability	[136,137,141]
	Genetic transfection of parental cells (nucleic acids/proteins)	miRNA/siRNA, mRNA, therapeutic proteins, targeting ligands	Precise loading; stable expression of defined cargo; preserves EV integrity; enables surface display of targeting/immune ligand	Efficiency depends on transfection and cell type, with possible effects on viability, limited control over copies per EV, and a risk of off-target effects	[124,136,142]
Post-loading (passive)	Simple co-incubation of isolated EVs with drugs	Small hydrophobic molecules (e.g., curcumin)	Technically easy; no special equipment; preserves EV morphology and surface markers	Often low loading; mainly suitable for lipophilic cargos; limited control of dose per EV	[133,134,135,136,137]
Post-loading (active-physical)	Electroporation	siRNA, miRNA, oligonucleotides and some small molecules	Increases loading of hydrophilic/charged cargos; widely used and relatively standardized	EV aggregation and membrane damage; cargo precipitation (esp. siRNA); protocol-dependent efficiency	[135,136,137,145]
	Ultrasonication	Small molecules (e.g., photosensitizers, hydrophobic drugs)	Higher loading than simple incubation; suitable for poorly soluble drugs; can promote deep lumen loading	May alter EV size, structure and surface proteins; risk of degradation with high energy; needs cargo-specific optimization	[135,136,144]
Post-loading (active-chemical)	Transfection reagents with isolated EVs	miRNA/ siRNA, plasmid DNA, antisense oligonucleotides	Mild conditions; generally preserve EV morphology; effective for nucleic acid; amenable to scaling	Contamination with free lipoplexes; hard to distinguish EV-bound from non-EV cargo; in vivo toxicity of reagent must be assessed	[136,143,145]
	Saponin-assisted loading	Hydrophilic small molecules, photosensitizers	Enhanced loading of hydrophilic cargos; simple and low-cost; combinable with other methods	High concentrations can cause irreversible damage and leakage; residual saponin may affect safety and biodistribution	[135,146]
	Calcium chloride-mediated transfection/ fusion	miRNA/ siRNA, plasmid DNA, other nucleic acids	Better nucleic acid loading than passive methods; relatively gentle on EV structure; simple and inexpensive	Variable efficiency and reproducibility; requires careful Ca^2+^ optimization; may alter surface charge and stability	[135,147]

### 5.2. EVs in Cancer Treatment

The potential of EVs as therapeutic tools in cancer treatment has garnered significant attention due to their ability to deliver targeted therapies with high specificity and minimal side effects. Numerous studies have highlighted the promise of engineered EVs in overcoming key challenges in oncology, such as drug resistance and tumor targeting, providing a new avenue for cancer treatment (Table 3).

EV-based therapeutics have also been investigated in non-CNS malignancies, including pancreatic ductal adenocarcinoma, breast cancer and chronic myeloid leukemia. In pancreatic cancer, engineered exosomes (iExosomes) delivering KRAS^G12D-targeting RNA interference (RNAi) have shown antitumor activity in preclinical models and have progressed to a first-in-human clinical trial, highlighting the feasibility of EXOs-based RNA therapeutics in patients [148]. In breast cancer, EXOs displaying targeting ligands such as the GE11 peptide or A15 have been used to deliver chemotherapeutic agents and miRNAs to EGFR- or integrin α_vβ_3-expressing tumor cells, resulting in reduced tumor growth and limited off-target effects in vivo [149,150]. Similarly, IL3-Lamp2b-engineered EXOs loaded with Imatinib or BCR-ABL siRNA have selectively targeted IL3 receptor-positive chronic myeloid leukemia cells and inhibited tumor growth in xenograft models [151].

These approaches fall under the broader category of EV surface engineering strategies, which are designed to improve targeting specificity and uptake. Surface engineering can be achieved through ligand or peptide display, antibody or aptamer conjugation, or genetic modification of membrane proteins such as Lamp2b fusion constructs. Such modifications enhance receptor-mediated recognition and are particularly valuable in neuro-oncology, where efficient engagement with the BBB/BBTB is essential for intracranial delivery. These examples illustrate general design principles for EV-based therapeutics in systemic oncology, including the use of surface ligands for selective uptake and the delivery of RNA-based or chemotherapeutic cargos (Table 3).

Overall, incorporating targeting ligands into EVs represents a promising strategy to enhance the delivery of therapeutic agents. This approach improves targeting efficiency to specific cells and tissues, including those shielded by biological barriers such as the BBB. An important future direction is the design of dual-specific, “logic-gated” EVs, in which the vesicle membrane is engineered to co-display distinct ligands, for example, a monoclonal antibody together with an aptamer recognizing an independent tumor-associated marker, thereby implementing an AND-type recognition that further increases tumor specificity and limits off-target uptake [155,156,157]. In addition, this strategy may help EVs evade immune clearance, increasing their potential as effective delivery systems.

In the treatment of GBM, EVs have shown significant potential (Table 3). Yang et al. investigated the therapeutic application of brain endothelial cell-derived EVs as nanocarriers for delivering siRNA targeting VEGF [138]. These engineered EVs effectively transported VEGF siRNA, which regulates tumor-induced angiogenesis. The siRNA silenced *VEGF* gene expression, inhibiting tumor growth in glioblastoma-astrocytoma U-87 MG cells and zebrafish xenografts, demonstrating a promising approach for targeted brain cancer therapy. These observations are in line with recent reports showing that engineered EVs can achieve BBB penetration and targeted delivery within GBM. This broadens the therapeutic landscape for EV-based interventions in the CNS [158,159,160].

A recent study further explored the use of EVs in GBM treatment through an innovative CRISPR-Cas9 gene editing approach [139]. A non-viral EV-based delivery system, known as Angiopep-2 (Ang) trans-activator of the transcription (TAT) peptide dual-modified EV (Ang/TAT-EVs), was developed to protect and transport Cas9 protein and single-guide RNA (sgRNA), enabling effective BBB penetration and precise tumor targeting. This system achieved gene editing efficiency in preclinical models while significantly reducing off-target effects. The primary therapeutic target identified was glutathione synthetase (*GSS*), a gene involved in ferroptosis regulation and radioresistance. *GSS* depletion sensitized tumor cells to radiotherapy by promoting ferroptosis, highlighting the potential of EVs as delivery platforms for targeted therapies in brain tumors [139].

Zhan Y. and colleagues observed the potential of EV delivery systems for targeted therapy by using miR-1208 as a tumor suppressor gene, which inhibits tumor growth and malignancy when downregulated in glioma tissues [140]. When loaded into EVs secreted by human bone marrow stromal cells (hBMSCs), miR-1208 crosses the BBB, acting as a tumor suppressor by inhibiting key pathways involved in glioma progression, by negatively regulating the expression of Methyltransferase-like 3 (*METTL3*) and suppressing the TGF-β pathway. Moreover, combining EVs loaded with miR-1208 with focused ultrasound (FUS) enhances their tumor-suppressive effects, providing a novel approach for glioma treatment [140].

EV-based therapies also show considerable potential in immunotherapy (Table 3). For example, loading EVs from dendritic cell-derived extracellular vesicles (DEVs) with cytokines such as IL-12 stimulates an anti-tumor immune response by recruiting immune cells to the tumor, such as CD8+ T-cells, NK-cells, and dendritic cells (DCs). This approach reduces immunosuppressive cells in the TME and promotes a Th1-dominant immune response that inhibits tumor growth and angiogenesis [152]. Importantly, EVs minimize the toxicity and side effects typically associated with traditional cytokine therapies, making them a safer and more effective option for treating brain tumors [152]. Furthermore, EVs loaded with CpG-STAT3 antisense oligonucleotides have been shown to activate immune cells, such as macrophages and DCs, leading to robust anti-tumor responses in the glioma microenvironment [154]. Likewise, EVs have been employed as carriers of tumor-specific neoantigens and vaccine components, including those directed against glioblastoma-associated mutations, thereby enhancing cytotoxic T cell infiltration and extending survival outcomes in both clinical and preclinical settings [161,162,163].

An innovative approach involves the use of genetically engineered mesenchymal stem cells (MSCs) to produce EVs loaded with therapeutic enzymes, such as yeast cytosine deaminase and uracilphosphoribosyl transferase (yCD::UPRT) [153]. These MSC-derived EVs, when combined with the prodrug 5-fluorocytosine (5-FC), enable selective tumor cell killing through a gene-directed enzyme prodrug therapy (GDEPT) mechanism. This therapeutic strategy has demonstrated encouraging efficacy in both in vitro and in vivo models, significantly inhibiting glioblastoma cell proliferation and underscoring its potential as a safer and more effective alternative to conventional treatments [153].

In the context of brain tumor treatment, it is also important to compare EV-based therapeutics with other nanocarrier platforms, particularly synthetic lipid nanoparticles (LNPs). LNPs have demonstrated remarkable clinical success as nucleic acid delivery systems and benefit from highly controllable composition, reproducible formulation and scalable manufacturing [164,165]. However, their use in neuro-oncology is still limited by issues such as systemic toxicity, inflammatory responses and suboptimal penetration of an intact BBB [165]. In contrast, EVs are endogenous vesicles with intrinsic biocompatibility, lower immunogenicity and a natural propensity to interact with specific cell types in the CNS, which may support more favorable biodistribution and immune tolerance. Moreover, EV membranes can incorporate complex combinations of surface proteins and lipids, as well as engineered targeting ligands, potentially enhancing BBB/BBTB crossing and tumor-specific uptake compared with non-biological carriers [166]. On the other hand, EV-based approaches face challenges that are less pronounced for synthetic systems, including vesicle heterogeneity, incomplete characterization of in vivo fate, limited cargo-loading efficiency and difficulties in large-scale, standardized production. Overall, EVs and LNPs should be viewed as complementary platforms: LNPs currently offer more mature and industrially scalable technologies, whereas EVs provide a biologically inspired alternative that may be particularly advantageous for precision delivery in the brain but still requires substantial optimization and clinical validation in neuro-oncology [164,165,166].

Rather than competing platforms, EVs and LNPs should therefore be viewed as complementary toolkits that can be tailored to distinct clinical scenarios and delivery challenges in neuro-oncology.

In terms of biocompatibility and safety, it is important to recognize that EVs are not intrinsically “non-immunogenic”, and their interaction with the host immune system critically depends on their cellular origin, surface composition, dose and route of administration [124,167]. EVs derived from mesenchymal stromal cells, DCs or other non-malignant sources have generally shown good tolerability in preclinical models and early-phase clinical studies [124,152,153,154,167], whereas tumor-derived EVs can carry immunosuppressive or pro-inflammatory molecules that may exacerbate disease or induce unwanted systemic effects [110,111,112,113]. Moreover, after systemic administration, EVs rapidly acquire a dynamic protein corona that can reshape their biodistribution, cellular uptake and immune recognition, with recent work demonstrating that corona composition critically modulates the pro-angiogenic and immunomodulatory activities of therapeutic EV preparations [125]. Similar source- and surface-dependent effects have been reported for synthetic nanomaterials, such as graphene oxide-loaded PLGA scaffolds, where increasing nanosheet content altered T-cell expansion and monocyte-macrophage differentiation in a dose-dependent manner despite the use of ostensibly “biocompatible” components [168]. These observations highlight the need for rigorous, source-specific safety assessment of EV-based products, including the analysis of off-target tissue accumulation, innate and adaptive immune activation, and potential long-term effects, before their widespread clinical implementation in neuro-oncology [124,125,167,168].

Beyond the intrinsic biological heterogeneity of EVs, technical variability in isolation and purification protocols has a major impact on their measured composition and functional properties [169,170]. Different workflows, such as differential and density-gradient ultracentrifugation, size-exclusion chromatography, polymer-based precipitation, and immunoaffinity capture, differ markedly in their ability to remove protein aggregates, lipoproteins, and non-vesicular nucleic acids, thereby enriching for distinct EV subpopulations [49,169,170]. As a consequence, studies employing different methodologies may report partially divergent EV cargo profiles and bioactivities, complicating cross-study comparisons and the identification of robust, reproducible EV-based biomarkers [49,170]. Complementary studies have also described semi-automated, GMP-oriented workflows for large-scale biomanufacturing of clinical-grade EV preparations and proposed practical quality-control and release frameworks to facilitate their standardization in translational and regulatory settings [171,172].

From a translational perspective, the current lack of harmonized, GMP-compatible procedures for large-scale EV production and purification contributes to batch-to-batch variation in particle number, size distribution and cargo composition, which directly affects dose definition, safety evaluation and regulatory approval [49,167]. Systematic head-to-head comparisons of isolation methods, adherence to MISEV reporting guidelines and the use of orthogonal characterization techniques (e.g., nanoparticle tracking analysis, electron microscopy and omics-based profiling) will be essential to reduce methodological bias and to enable the development of clinically reliable EV-based diagnostics and therapeutics [49,167,170].

Despite these advances in using EVs as therapeutic vehicles, particularly in preclinical models of GBM and other brain tumors, several critical challenges persist that hinder their widespread clinical application. EV heterogeneity, stemming from different biogenesis pathways and dynamic cargo compositions, complicates their standardization for therapeutic use. Current cargo-loading strategies, such as electroporation or incubation with donor cells, often exhibit suboptimal efficiency, necessitating the administration of large EV quantities that may elevate toxicity risks. Moreover, scaling up EV production for clinical applications presents logistical and technical limitations. An additional layer of complexity is represented by the formation of a protein corona on the EV surface, as discussed above, which can further modulate biodistribution, immune evasion and off-target interactions [125]. Overcoming these multifaceted obstacles is essential for the clinical translation of EV-based therapies. Continued progress in EV biology, bioengineering, and biomanufacturing is expected to unlock the full potential of EVs as next-generation drug delivery platforms in oncology and regenerative medicine.

## 6. Conclusions

EVs are emerging as powerful platforms for therapeutic innovation in the treatment of brain tumors, including GBM, owing to their intrinsic ability to deliver molecular cargo across cellular membranes and physiological barriers such as the BBB. Beyond their diagnostic potential as liquid biopsy biomarkers, EVs offer a unique vehicle for the delivery of targeted therapeutics, facilitating precision oncology strategies in neuro-oncology. However, their dual functional nature complicates their therapeutic use: while EVs derived from non-malignant cells may exert tumor-suppressive effects, especially in early disease stages, tumor-derived EVs often facilitate cancer progression by modulating the tumor microenvironment, promoting immune evasion, and reprogramming normal cells to support malignancy. This dual role highlights the complexity of EV biology in the context of brain tumors.

Advances in genetic engineering, cargo customization, and EV-based delivery systems offer significant promise for the future of brain tumor treatment. However, major challenges remain, including the need for enhanced targeting specificity, efficient and reproducible cargo incorporation, and scalable GMP-compliant production protocols. A deeper understanding of the molecular mechanisms underpinning EV biogenesis, cargo sorting, and intercellular signaling is critical to unlocking their full potential as a transformative tool in brain tumor therapy.

## Figures and Tables

**Table 1:** Extracellular vesicle-associated molecular regulators in Glioblastoma: functional roles in tumor progression, immune modulation, and microenvironmental remodeling.

Molecular Group	Molecular Regulator	Type	Biological Function	References
Heat shock proteins (Hsps)	Hsp27	Tumor promoter	Enhances tumor growth and cancer cell proliferation	[79,80]
	Hsp47	Tumor promoter	Promotes tumor growth, invasiveness, and angiogenesis	[81,82]
	Hsp70	Tumor promoter	Promotes cancer cell proliferation, migration, and invasion	[83,84]
	Hsp90	Tumor promoter	Increases cancer cell motility and invasion	[85,86]
microRNA (miRNAs)	miR-9	OncomiR	Stimulates angiogenesis	[87,88,89]
	miR-451	OncomiR	Promotes tumor growth and progression	[90,91]
	miR-21	OncomiR	Promotes tumor growth and progression and inhibits immune cell activity	[90,91,92]
	miR-10a	OncomiR	Induces immunosuppression	[93]
	miR-29a	OncomiR	Enhances immunosuppression and proliferation	[94]
	miR-1246	OncomiR	Supports immune evasion	[95]
	miR-1	Tumor suppressor	Inhibits angiogenesis and invasion	[76]
	miR-124	Tumor suppressor	Reprograms GBM cells to reduce harmful metabolites	[96]
	miR-101-3p	Tumor suppressor	Inhibits cell proliferation, migration and invasion	[97]
	miR-944	Tumor suppressor	Suppresses angiogenesis, cell proliferation, and migration	[98]

**Table 3:** Engineered extracellular vesicles (EVs) as nanotherapeutic systems in brain and non-brain cancers.

	Cancer Type	EVs Cargo Molecules	Therapeutic Target	References
NON-BRAIN CANCER	Pancreatic ductal adenocarcinoma (PDAC) and Metastatic pancreatic adenocarcinoma	KRAS^G12D^ RNAi	KRAS^G12D^ mutation	[148]
		KRAS^G12D^ siRNA		NCT03608631 (ClinicalTrials.gov)
	Breast cancer	GE11 peptide or EGF	EGFR-expressing cancer cells	[149]
		A15 ligand,	Integrin α_v_β_3_	[150]
		Doxorubicin (Dox), Cho-miR159	TCF-7 gene	
	Chronic Myeloid Leukemia	IL3-Lamp2b fusion protein	IL3- receptor -expressing cancer cells	[151]
		Imatinib or BCR-ABL siRNA	BCR-ABL fusion oncogene	
BRAIN CANCER	Glioblastoma (GBM)	VEGF siRNA	VEGF (angiogenesis inhibition)	[138]
		CRISPR-Cas9 sgRNA	Glutathione synthetase (GSS)	[139]
		IL-12 in DEVs	Stimulate an anti-tumor immune response	[152]
		Cytosine deaminase and uracilphosphoribosyl transferase (yCD::UPRT)	Prodrug 5-fluorocytosine (5-FC) conversion to cytotoxic 5-FU	[153]
	Glioma	miR-1208	Downregulating METTL3 and suppressing TGF-β pathway	[140]
		CpG-STAT3 antisense oligonucleotides	Activate immune cells	[154]

## Data Availability

No new data were created or analyzed in this study. Data sharing is not applicable to this article.

## Abbreviations

APCs, antigen-presenting cells; BBB, blood-brain barrier; BBTB, blood-brain tumor barrier; BCSFB, blood-cerebrospinal fluid barrier; CBTRUS, Central Brain Tumor Registry of the United States; cfDNA, cell-free DNA; CNS, central nervous system; CRISPR-Cas9, clustered regularly interspaced short palindromic repeat-CRISPR-associated protein 9; CSF, cerebrospinal fluid; CT, computed tomography; CTCs, circulating tumor cells; DCs, dendritic cells; DEVs, dendritic cell-derived extracellular vesicles; EVs, extracellular vesicles; EXOs, exosomes; FUS, focused ultrasound; GBM, glioblastoma multiforme; GDEPT, gene-directed enzyme prodrug therapy; GLOBOCAN, Global Cancer Observatory database; GMP, good manufacturing practice; hBMSCs, human bone marrow stromal cells; HDI, Human Development Index; Hsps, heat shock proteins; ISEV, International Society for Extracellular Vesicles; LNPs, lipid nanoparticles; miRNAs, microRNAs; MISEV, Minimal Information for Studies of Extracellular Vesicles; MRI, magnetic resonance imaging; MSCs, mesenchymal stem cells; MVs, microvesicles; NK, natural killer (cells); PDAC, pancreatic ductal adenocarcinoma; PD-L1, programmed death-ligand 1; PLGA, poly (lactic-co-glycolic acid); RF-EMF, radiofrequency electromagnetic fields; RNAi, RNA interference; sEVs, small extracellular vesicles; TGF-β, transforming growth factor-β; TME, tumor microenvironment; TRAIL, tumor necrosis factor-related apoptosis-inducing ligand; VEGF, vascular endothelial growth factor; WHO, World Health Organization; yCD::UPRT, yeast cytosine deaminase/uracil phosphoribosyltransferase.
